# Does a Transcriptionally Active HPV Infection Affect the Invasiveness of Pituitary Neuroendocrine Tumors? A Case Series Study of 60 Patients in Krakow, Poland

**DOI:** 10.3390/cancers17040684

**Published:** 2025-02-18

**Authors:** Anna Krzentowska, Beata Biesaga, Ryszard Czepko, Dariusz Adamek, Anna Merklinger-Gruchała, Filip Gołkowski

**Affiliations:** 1 Department of Endocrinology and Internal Medicine, Andrzej Frycz Modrzewski Krakow University Medical College, 30-705 Kraków, Poland; 2 Department of Medical Biology, Andrzej Frycz Modrzewski Krakow University Medical College, 30-705 Kraków, Poland; 3 Department of Neurosurgery, Andrzej Frycz Modrzewski Krakow University Medical College, 30-705 Kraków, Poland; rczepko@uafm.edu.pl; 4 Department of Neuropathology, Collegium Medicum, Jagiellonian University, 31-531 Kraków, Poland; mnadamek@cyf-kr.edu.pl; 5 Department of Pathomorphology, Collegium Medicum, Jagiellonian University, 31-531 Kraków, Poland; 6 Department of Bioinformatics and Public Health, Andrzej Frycz Modrzewski Krakow University Medical College, 30-705 Kraków, Poland; amerklinger@uafm.edu.pl

**Keywords:** HPV infection, pituitary adenoma, invasiveness, Knosp scale, Hardy scale

## Abstract

Background/Objective: Pituitary neuroendocrine tumors (PITNETs) often show a tendency towards invasive behavior, i.e., an invasion towards the cavernous sinuses or destruction of the sella turcica. In the present study, we analyzed whether a transcriptionally active HPV infection affects the invasiveness of pituitary tumors. Methods: Sixty patients with different phenotypes of PITNETs who underwent neurosurgery were studied. The obtained postoperative material was analyzed histopathologically. For each patient, formalin-fixed paraffin-embedded blocks were cut into ultra-thin slices and two to three of them were designated for DNA extraction, while one was used for histological slides. Based on the isolated DNA, the presence of DNA from individual HPV types was determined by the real-time detection polymerase chain reaction using the REALQUALITY RQ-Multi HPV Detection reagent kit (AB ANALITICA, Italy). P16 protein expression was assessed by immunohistochemical staining on the histological slides. A transcriptionally active infection with individual HPV types was distinguished when HPV DNA and P16 protein overexpression were detected simultaneously for a given tumor. Results: In the group of the 60 analyzed PITNETs, a transcriptionally active high-risk HPV infection was detected in a subset of 11 tumors (18.3%). This infection was associated with a significantly lower probability of tumor invasiveness, measured on both the Knosp (OR = 0.11, 95% CI: 0.02–0.58) and Hardy scales (OR = 0.12 95% CI: 0.024–0.56). Conclusions: Further studies are needed to confirm the prevalence of transcriptionally active HPV infections in pituitary adenomas and the role of these infections in the invasiveness of pituitary tumors.

## Simple Summary

Invasiveness of pituitary neuroendocrine tumors (PITNETs) is a major cause of recurrence and the risk of reoperation. Therefore, identification of specific biomarkers for the diagnosis and effective treatment of invasive PITNETs is of a great clinical importance. The aim of our retrospective study was to assess the potential of transcriptionally active HPV infection as a prognostic factor for PITNET invasiveness expressed in Knosp and Hardy scales. Among 60 PITNETs, a transcriptionally active high-risk HPV infection was detected in 11 tumors (18.3%). This infection was associated with a significantly lower probability of tumor invasiveness, measured on the Knosp and Hardy scales. Our results suggest that this transcriptionally active HPV infection may influence the invasiveness of pituitary adenomas; however, further studies are needed to confirm these results.

## 1. Introduction

Pituitary neuroendocrine tumors (PITNETs) account for approximately 16% of all primary brain tumors and for almost 25% of benign primary brain tumors [1]. The primary treatment of PITNETs remains a neurosurgical procedure, i.e., a transsphenoidal resection. Due to the invasion of pituitary tumors into the surrounding structures, their complete resection is often not possible. In the absence of a surgical cure, conservative treatment with first- and then second-generation somatostatin analogues is used. The presence of a pituitary tumor leads to complications related to its endocrine function, as well as to symptoms due to the mass effect and the fact that the tumor has spread to the surrounding structures.

Clinically, PITNETs are divided into hormonally inactive and active tumors, i.e., those secreting in excess such hormones as ACTH (adrenocorticotropic hormone), GH (growth hormone), PRL (prolactin), TSH (thyroid-stimulating hormone), LH (luteinizing hormone), and FSH (follicle-stimulating hormone). Hormonally inactive PITNETs are the most frequent pituitary tumors [2], and their diagnosis is often based on symptoms of mass effect, such as the most commonly noted—headaches and visual field abnormalities [3]. On the other hand, from a neurosurgical point of view, tumors are divided into invasive and non-invasive using two scales, i.e., the Knosp scale [4], taking into account the penetration of the tumor towards the cavernous sinuses, and the Hardy scale [5], assessing the degree of destruction of the sella turcica. Now, however, due to the important role of transcription factors in the development of these tumors, the World Health Organization (WHO) in 2017 proposed the division of PITNETs into PIT 1-lineage tumors (PIT-1; pituitary-specific POU-class homeodomain transcription factor), TPIT (T-box family member TBX19)-lineage tumors, SF1 (SF-1, steroidogenic factor)-lineage tumors, and tumors without a distinct cell lineage [6]. In 2022, the WHO introduced a modification to the above classification: the category of PIT-1-positive plurihormonal tumors was replaced by two clinically distinct PITNETs: immature PIT-1-lineage and mature PIT-1 plurihormonal-lineage tumors [7]. The assessment of tumor proliferation and invasion was recommended to identify tumor aggressiveness. A number of researchers are currently searching for factors affecting the development of PITNETs [8,9,10,11]. For PITNETs, tumor invasiveness is a major cause of recurrence and the risk of reoperation. Therefore, the identification of specific biomarkers for the diagnosis and effective treatment of an invasive PITNET is of a great clinical importance. At present, markers are being sought that affect tumor invasiveness and thus indicate the possibility of complete tumor resection.

Human papillomavirus (HPV) is among the most prevalent viral infections worldwide, affecting millions annually [12]. High-risk HPV (hrHPV) infection is a well-established risk factor for certain anogenital cancers, including cervical, vulvar, penile, and anal cancers. While the role of HPV in these cancers is well established, its involvement in carcinogenesis at other sites, such as the breast, lung, or pituitary gland, remains inconclusive and warrants further scientific investigation. Detecting HPV in pituitary tumors could provide valuable insights into their etiology and behavior, potentially influencing diagnostic and therapeutic strategies. For instance, identifying HPV in these tumors might suggest a viral contribution to tumor development, opening avenues for targeted therapies or preventive measures. Moreover, understanding the presence of HPV in pituitary tumors could aid in prognostication, similar to its role in oropharyngeal cancers [13]. Currently, there is insufficient evidence to suggest a connection between HPV infection and the development of pituitary tumors. The first and only study on this topic was conducted by researchers from China [14]. In a group of 60 patients with pituitary adenomas, they found, using nested PCR with primers specific for HPV16, the prevalence of this HPV type in 29 patients (48.3%). They also revealed that HPV16 presence was significantly higher in invasive (70.0%) than in non-invasive pituitary adenomas (26.7%). However, these results should be confirmed in other studies. Therefore, considering the significant gap in global literature regarding the occurrence of transcriptionally active hrHPV infection and its relevance to the invasiveness of pituitary tumors, the objective of this study was to evaluate transcriptionally active HPV infection in pituitary tumors, as well as to analyze the correlation between the presence of this infection and the invasiveness of pituitary tumors as expressed on the Knosp and Hardy scales.

## 2. Materials and Methods

### 2.1. Patient Population

The study included a group of 60 patients who underwent transsphenoidal surgery via the transnasal approach at the Hospital of St. Raphael in Krakow in the period from February 2022 to May 2023. Pituitary adenomas were subsequently confirmed by histopathology (HP). Each patient gave informed consent for the collection of tumor tissue for examination. Patients who did not have a pituitary adenoma confirmed on the HP examination after surgery were excluded from the study. The database contained clinical and demographic information, such as gender and age at diagnosis. Each patient had a magnetic resonance imaging (MRI) scan of the head or a pituitary-targeted MRI scan prior to surgery; in individual cases, a head CT scan was performed due to a contraindication of MRI. Based on the MRI scan, the tumor was measured in 3 dimensions, i.e., AP, ML, and CC (cor × sag × cc), and the tumor volume was calculated. In addition, tumor invasion into the cavernous sinuses was assessed using the Knosp scale, while the erosion of the sellar floor and invasion of the sphenoidal sinus were assessed according to the Hardy scale. On the Knosp scale, grades 3 and 4 were treated as invasive tumors, while grades 1 and 2 were classified as non-invasive tumors. Similarly, on the Hardy scale, grades 1 and 2 were classified as non-invasive and tumors, and grade 3 and above were defined as invasive tumors. The patients were referred to a neurosurgeon for symptoms such as headaches, dizziness, tinnitus, sudden visual disturbances, and sudden ptosis. The patients underwent transsphenoidal resection of the pituitary tumor in a single neurosurgical center.

For each patient, on the basis of formalin-fixed paraffin-embedded (FFPE) tumor specimens, the expression of the pituitary hormones (ACTH, GH, PRL, TSH, LH, FSH) and the immunohistochemical evaluation in a search for the transcription factors (PIT-1, TPIT, SFi1) were performed. The hormonal activity of the tumor was assessed (0—none, 1—hormonally active), while on the basis of the presence of positive expression of the transcription factors, the tumors were classified according to the 2022 WHO classification terminology.

### 2.2. Materials

For each patient, formalin-fixed and paraffin-embedded (FFPE) blocks were cut into ultra-thin slices at the Consilio Diagnostics Facility in Krakow, and 2–3 of them were designated for DNA extraction and 1 for histological slides. To avoid cross-contamination, a new, sterile microtome blade was used for each tissue sample.

### 2.3. DNA Extraction

DNA was extracted from 2–3 sections of FFPE, using the ReliaPrep FFPE gDNA Miniprep System (Promega Corporation, Madison, WI, USA) according to the manufacturer’s suggestions, with our modification added. Deparaffinization was carried out using mineral oil heated to 80 °C. Subsequently, a lysis buffer was added to the samples, and after centrifugation, two distinct phases were observed: an aqueous phase containing the tissue and an oil phase containing dissolved paraffin. Proteinase K was introduced into the aqueous phase, and the samples were incubated overnight (our modification) at 56 °C. The samples were then incubated for 1 h at 80 °C, treated with RNase A, and combined with BL buffer and 96% ethanol. Following centrifugation, the entire aqueous phase containing DNA was transferred to a binding column, washed twice, and finally eluted. DNA concentration and purity (assessed by the A260/280 and A260/230 ratios) were measured using a Biophotometer Plus (Eppendorf, Wesseling-Berzdorf, Germany). The samples were stored at −20 °C until needed.

### 2.4. HPV Genotyping Assay

Based on the isolated DNA, the presence of DNA from individual HPV types was checked by the real-time detection polymerase chain reaction (qPCR) using the REALQUALITY RQ-Multi HPV Detection reagent kit (AB ANALITICA, Padova, Italy). This kit allows for the detection of 20 HPV types from the high-risk subgroup (hrHPV—separately, HPV16 and 18, and one or more of the following genotypes: 31, 33, 35, 39, 45, 51,52, 56, 58, 59, 66, 68) and 8 subtypes from the low-risk subgroup (lrHPV—separately, HPV6 and/or 11, one or more of the following genotypes: 40, 42, 43, 44, 55, 83 and one or more of the following genotypes: 26, 53, 67, 70, 73, 82).

In the REALQUALITY RQ-Multi HPV Detection reagent kit a ready-to-use master mix is provided, containing a cocktail of hydrolysis probes labeled with four different fluorochromes: FAM for the hrHPV group, JOE for HPV 16, Cy5 for HPV 18, and ROX for the control gene. In the reaction, the human beta-globin gene is also detected, which allows for monitoring both the extraction and amplification processes. A positive control, which allows for the monitoring of fluorescence detection across all the channels, is also supplied by the manufacturer and was included in each run. Molecular-grade water, as a negative control, was added. The assay was performed according to the manufacturer’s protocol on the BioRadCFX96 platform (BioRad, Hercules, CA, USA). Thermal cycling was initiated with 2 min incubation at 50 °C, followed by 10 min denaturation at 95 °C. Then, 40 cycles of 95 °C for 20 s and 65 °C for 60 s were applied. For each DNA sample, the reaction was conducted in triplicate. The presence of viral DNA was confirmed when, in all the three replicates, at least one HPV amplification curve crossed the established threshold line, as defined by the manufacturer’s guidelines, with a corresponding cycle threshold (Ct) value of <40, and the Ct for the internal control gene was ≤34. A sample was classified as negative if, in at least one replicate, no HPV amplification curve crossed the threshold.

### 2.5. Immunohistochemical P16 Staining

Based on the prepared histological slides, immunohistochemical staining was performed for the presence of the P16 protein expression. P16 staining was carried out using the CINtec p16INK4a Histology Kit (Roche Diagnostics, Mannheim, Germany), following the manufacturer’s protocol. Briefly, 4 µm-thick sections of FFPE HNSCC tissues were deparaffinized and rehydrated through a series of xylenes and alcohols. After antigen retrieval (96 °C, 10 min) and blocking of exogenous peroxidases (5 min), the sections were incubated with the primary anti-p16 antibody (clone E6H4) at room temperature for 30 min, followed by a 30 min incubation with the visualization system. P16 was visualized using DAB (3,3′-diaminobenzidine), and nuclear counterstaining was performed with hematoxylin. A cervical cancer tissue with known p16 overexpression served as a positive control, while a negative control was prepared by omitting the primary antibody.

**Figure 1:**
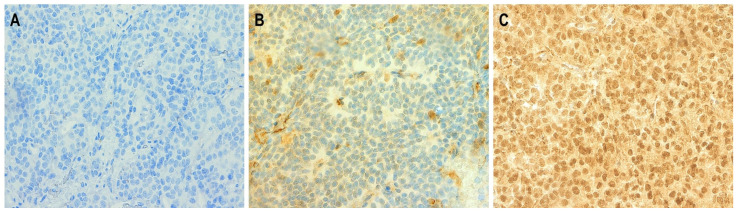
Microphotographs related to immunohistochemical staining for the presence of P16 expression. No positive staining—absence of P16 expression (**A**). Visible cells showing P16 expression; however, staining does not meet the criteria for positivity, as staining is present in less than 75% of tumor cells (**B**). Positive staining for P16—staining present in more than 75% of tumor cells (**C**). Magnification: ×200.

The stained sections were independently evaluated by two researchers. P16 overexpression, according to the paper by Lewis et al. [15], was considered if moderate to strong and diffuse staining (nuclear and/or cytoplasmic) was present in more than 75% of the tumor cells or >50% of such cells were found in the preparation and at the same time there was continuous staining in >25% of the tissue (Figure 1). All other staining patterns, including focal, weak, or absent staining, were classified as P16 negative (Figure 1).

### 2.6. Identification of a Transcriptionally Active HPV Infection

Transcriptionally active infections with individual HPV types were distinguished when the HPV DNA and P16 protein overexpression were detected simultaneously for a given tumor. In other cases, (HPV DNA+/P16−, HPV DNA−/P16+, and HPV DNA−/p16−), the tumors were classified as HPV negative.

### 2.7. Statistical Analysis

Descriptive statistics were used to determine the mean and median level for continuous variables. The relationships between the categorized variables were analyzed using the Pearson’s chi-squared test.

A univariate logistic regression model was run with tumor invasiveness (on both the Knosp and Hardy scales) as a dichotomous outcome variable and transcriptionally active hrHPV infection as a predictor. The aim of the next step of the analysis was to create a multivariate logistic regression model, with transcriptionally active hrHPV infection as the main predictor, adjusted by potential confounders. The selection of independent variables to be included in this model was based on multicollinearity analyses. For this purpose, we relied on principal component analyses with varimax rotation, which extracted mutually correlated groups of factors. The representatives of these groups were selected, which were then included in the multivariate logistic regression (full) model as potential covariates.

Finally, the backward stepwise regression procedure was applied to test the independent variables in the multivariate logistic regression (final) model. The statistical significance of the model parameters was assessed using the Wald test. The model quality was assessed with the Akaike information criterion (AIC) and pseudo-R^2^ measures such as the Cox–Snell and Naglekerke coefficients.

Calculations were performed using Statistica v.13.3. A *p*-value of less than 0.05 was considered statistically significant.

## 3. Results

### 3.1. Characteristics of the Patient Group

A total of 60 patients with pituitary adenoma were included in the study (Table 1). The mean age of patients was 57.9 ± 1.7 years (SE). The group consisted mainly of men (58.3%) and patients with gonadotrophic PiTNETs. Among the 60 pituitary tumors, 10 were hormonally active, showing positive expression for ACTH, hGH, or PRL. Tumor invasiveness, assessed by the Knosp scale (grades 3 and 4), was noticed in 33 cases (55.9%), while by the Hardy scale (grade above 3) it was noticed in 48 tumors (87.5%).

Invasive tumors (in both the Knosp and Hardy scales) were characterized by larger size and volume (Table 2). These tumors were significantly larger in all three dimensions—anterior–posterior (AP), mediolateral (ML), and craniocaudal (CC). Other analyzed demographic and histopathological parameters were not associated with tumor invasiveness on either the Knosp or the Hardy scale.

### 3.2. The Prevalence of HPV DNA

In the group of 60 PITNETs, the presence of high-risk HPV DNA was detected in 13 tumors (21.7%) (Table 3). Within this subgroup, HPV16 DNA was predominantly found in the majority of tumors (n = 8). A single HPV16 infection was noted in seven (54.5%). One tumor exhibited a dual infection with HPV16 and one or more other hrHPV types (31, 33, 35, 39, 45, 51, 52, 56, 58, 59, 66, 68). In four additional PITNETs, the presence of one or more other hrHPV types was found (31, 33, 35, 39, 45, 51, 52, 56, 58, 59, 66, 68). Only one PITNET showed the presence of HPV18 DNA, and this was a co-infection with one or more other hrHPV types (31, 33, 35, 39, 45, 51, 52, 56, 58, 59, 66, 68). A statistically significant correlation was found between the presence of hrHPV DNA and tumor invasiveness according to the Knosp scale and Hardy scale (Table 4). The majority of invasive PITNETs lacked transcriptionally active hrHPV infections, whereas in non-invasive tumors these percentages were significantly higher. None of the evaluated demographic (age, gender), clinical (tumor size, hormonal activity), or histopathological parameters (expression of transcription factors PIT-1, TPIT and SF1, P16) had a significant impact on the frequency of hrHPV DNA detection.

Among the 60 analyzed PA cases, a low-risk HPV infection was detected in 26 tumors (43.3%), with the majority of these infections involving one or more of the following genotypes: HPV 26, 53, 67, 70, 73, or 82 only (n = 14) (Table 3). No significant effect of the lrHPV DNA presence was observed in the other analyzed demographic (age, gender), clinical (tumor size, hormonal activity), or histopathological parameters (expression of transcription factors PIT-1, TPIT and SF1, P16, presence of hrHPV).

### 3.3. Immunoexpression of P16

In the analyzed cohort of 60 PITNETs, overexpression of the P16 protein was observed in 21 cases (35.0%) (Table 4). No significant association was found between the frequency of P16 overexpression and tumor invasiveness as assessed by the Knosp and Hardy scales. Similarly, no statistically significant associations were noticed between the distribution of tumors with and without P16 overexpression and the analyzed demographic variables (age, gender), clinical factors (tumor size, hormonal activity), or histopathological parameters (expression of transcription factors PIT-1, TPIT, and SF1).

### 3.4. Transcriptionally Active Infection of hrHPV

In the group of 60 analyzed PITNETs, a transcriptionally active hrHPV infection (a simultaneous presence of P16 overexpression and viral DNA in the tumor) was detected in 11 tumors (18.3%) (Table 4). Among these, six cases involved a single HPV16 infection, while four cases were associated with other hrHPV types (31, 33, 35, 39, 45, 51, 52, 56, 58, 59, 66, 68). One tumor exhibited a co-infection of HPV16 and one or more hrHPV subtypes from group 31, 33, 35, 39, 45, 51, 52, 56, 58, 59, 66, or 68. Additionally, two tumors demonstrated the presence of hrHPV DNA but lacked P16 overexpression, and these ones were classified as negative for a transcriptionally active hrHPV infection. Detailed characteristics of 11 PITNETs with transcriptionally active hrHPV infection are presented in Table 5.

A statistically significant impact of transcriptionally active hrHPV infections on tumor invasiveness, as assessed by the Knosp and Hardy scales, was noticed (Table 4). The presence of transcriptionally active hrHPV infection was associated with a lower prevalence of invasive vs. non-invasive tumors on the Knosp scale (18.2% vs. 81.8%, respectively), and the lack of transcriptionally active viral infection was related to a higher prevalence of invasive vs. non-invasive tumors (66.0% vs. 34.0%, respectively; *p* = 0.01). A similar pattern was noted for the Hardy scale, i.e., although among PITNETs with transcriptionally active hrHPV infection the percentages of patients with invasive and non-invasive tumors were comparable (54.6% vs. 45.5%, respectively), this type of infection was still related to a higher prevalence of invasive vs. non-invasive tumors (89.4% vs. 10.6%, respectively; *p* = 0.02). No statistically significant associations were noticed between the distribution of tumors with or without P16 overexpression and the analyzed demographic variables (age, gender), clinical factors (tumor size, hormonal activity), or histopathological parameters (expression of transcription factors PIT-1, TPIT, and SF1).

### 3.5. Multivariate Logistic Regression (Full) Models

Multicollinearity analysis preceding multivariate modeling allowed for the identification of groups of intercorrelated variables. Features strongly correlated with tumor volume included tumor dimensions and maximum tumor size, which were not included in the next stage of variable selection for the multivariate regression model, i.e., principal component analysis. This analysis further showed that tumor volume was characterized by a high factor loading (>0.7) for one of the components (factor group), similar to the main predictor (transcriptionally active hrHPV infection) representing this factor group, and was ultimately not included in the multivariate model to avoid multicollinearity issues.

Multivariate logistic regression (full) models showed that tumor invasiveness (on both the Knosp and Hardy scales) was related to transcriptionally active hrHPV infection (Table 6). The other variables, such as age, gender, hormonal activity, PIT-1, and TPItT, were not significantly associated with the outcome (*p* > 0.05). After adjustment to the patient’s age and gender, hormonal activity, PIT-1, and TPIT, exposure to transcriptionally active hrHPV infection diminished the probability of tumor invasiveness by 96.6% (measured on the Knosp scale, OR = 0.03, 95% CI: 0.00–0.48) and by 93.7% (measured on the Hardy scale, OR = 0.06, 95% CI: 0.01–0.44).

The final models showed that the only significant predictor of tumor invasiveness was transcriptionally active hrHPV infection (*p* < 0.05), and it was the only factor that remained in the model after applying the backward stepwise logistic regression procedure. The presence of this infection was associated with a significantly lower probability of tumor invasiveness, measured on both the Knosp (OR= 0.11, 95% CI: 0.02–0.58) and Hardy scales (OR = 0.12 95% CI: 0.02–0.56).

## 4. Discussion

In this study, in the group of 60 patients with PITNETs we detected transcriptionally active hrHPV infection in 11 of 60 cases (18.3%). In contrast, Zheng et al. [14], also in the group of 60 PITNETs, reported a significantly higher percentage of HPV16 positivity. This discrepancy may stem from differences in the methods used to assess viral presence and from variations in the clinical characteristics of the patient groups. For hrHPV genotyping, we employed qPCR with the Real Quality RQ-Multi HPV Detection kit (AB ANALITICA, Italy) and P16 immunoexpression, a method, to our knowledge, first used in PITNETs. The kit was selected based on its validation by Iacobellis et al. [16], which compared its clinical efficacy to the Hybrid Capture 2 (HC2) assay. They demonstrated that the clinical sensitivity and specificity of the REALQUALITY RQ-HPV assay for detecting ≥CIN2 were non-inferior to HC2. However, it should be noted that HPV DNA reflects the presence of an ongoing infection but does not confirm whether the infection is transcriptionally active. A transcriptionally active HPV infection can be indirectly assessed by P16 overexpression, which is considered to be a cellular response to the degradation of pRb by the HPV E7 oncoprotein [17]. Contrary to us, Zheng et al. [14] used nested PCR combined with HPV16E6 immunoexpression to detect HPV16 infection. It should be emphasized, however, that nested PCR is known for high false-positive rates. Bozic et al. [18] reported that while nested PCR detected HPV DNA in 22% of samples, single PCR and real-time PCR did not detect HPV in any of 50 samples from head and neck cancer tissues. Zheng et al. [14] also used HPV16 E6 immunostaining, but there is no validated protocol for E6 immunostaining, including antibody type, epitope retrieval method, or scoring system, which raises questions about the reliability of their findings. Furthermore, it is unclear whether the anti-HPV16 E6 antibody used was validated through methods like Western blotting or immunofluorescence. Another factor contributing to the differences between our findings and those of Zheng et al. [14] may be the clinical characteristics of the patient groups. Zheng’s study included 30 patients with growth hormone-secreting tumors (GH-PAs) and 30 with hormonally inactive pituitary tumors (NFPAs), whereas our study had 50 NFPAs and 10 GH-PAs. Our GH-PAs were more heterogeneous, including corticotroph, lactotroph, thyrotroph, and plurihormonal PITNETs, which may have influenced the results. Both studies assessed invasiveness using the Knosp scale, with Zheng reporting 30 invasive and 30 non-invasive tumors, while our study included 33 invasive and 25 non-invasive tumors on the Knosp scale, and 48 invasive and 10 non-invasive tumors on the Hardy scale. Notably, the average age of patients in our study differed from Zheng’s. In our study, the average age for invasive tumors was 57.4 years (Knosp) and 58.4 years (Hardy), while non-invasive tumors averaged 58.4 years (Knosp) and 55.0 years (Hardy). Therefore, it should be state that our study group was more diverse, including different types of PITNETs, which could account for differences in the results. Zheng’s study focused exclusively on hormonally inactive and GH-secreting tumors, which tend to be larger and more invasive.

Our study demonstrated, according to our best knowledge, for the first time worldwide that among the demographic (age and gender), clinical (hormonal activity), and histopathological (PIT1, TPIT, transcriptionally active hrHPV infection) participant characteristics, the only factor that significantly affected the tumor invasiveness was transcriptionally active hrHPV infection. The results of the final models allowed us to assess that individuals exposed to transcriptionally active hrHPV infection had from 88% (on the Hardy scale) to 89% (on the Knosp scale) lower probability of having an invasive tumor compared to the individuals who were unexposed (Table 6). This hypothesis may be supported by the results concerning patients with HN, particularly oropharyngeal squamous cell carcinoma. In this group, numerous clinical studies and meta-analyses ([13]—review) have shown that patients with HPV16 infection have a better prognosis. In the group of HNC patients infected with HPV16, the virus has been shown to influence circulating immune cells as well as tumor-specific immune cells in the tumor microenvironment. Few studies have identified HPV-specific T cells in the blood of patients with hrHPV infections [19,20]. Additionally, specific HPV antibodies are detected in the plasma of infected individuals, which are rarely found in patients with HPV-negative tumors. Notably, CD4+ and CD8+ T cell responses are directed against all HPV E proteins (E1, E2, E4, E5, E6, and E7) [21]. Several studies have evaluated immune infiltrates in HPV+ HNC tumors and compared them to HPV-negative cancers. In these studies, significant differences in the composition of the tumor microenvironment (TME) between these two cancer types have been found [22,23]. Overall, HPV+ HNC displays several features associated with a more inflammatory or “hotter” TME, such as increased B cell infiltrates and higher frequencies of PD-1+ CD8+ tumor-infiltrating lymphocytes (TIL), type 1 helper (TH1) CD4+ T cells, TH17 CD4+ T cells, and follicular helper (TFH) CD4+ T cells [24]. However, in the case of PITNETs, only one study has assessed the impact of an hrHPV infection on tumor invasiveness, with results contrary to those obtained by us. Zheng et al. [14], in the group of 60 human PITNETs, found a statistically significant higher occurrence of HPV16 DNA in invasive tumors (70%) compared to non-invasive tumors (26.7%). Similar to our study, invasiveness in their study was assessed using the Knosp scale. Moreover, individual studies on the immune system activity in PA patients challenge the hypothesis that hrHPV stimulates the immune system and thus reduces the invasiveness of these tumors. Zhou et al. [25] demonstrated that patients with GH-PAs exhibited higher levels of B cells and CD8 T cell infiltration in the tumor. At the same time, GH-PAs were more frequently invasive and independently correlated with a shorter progression-free survival. Similarly, Zheng et al. [14] found an increased TLR3 mRNA expression—a receptor protein recognizing foreign antigens—in invasive PA samples. These results are in contradiction to the hypothesis of immune system stimulation by hrHPV. Taking this into account, it seems that further studies should focus on analyzing the relationship between TME biomarkers and transcriptionally active hrHPV infection. It appears that the discrepancies regarding the influence of hrHPV may stem from differences in methods for assessing viral infections, as well as from variations in the clinical characteristics of the studied groups, as presented in the first part of this discussion. Our study has some limitations related to the fact that it is a case study without a control group. However, in comparative studies, the ideal control group would consist of individuals without pituitary tumors. Surgeons typically aim at preserving the healthy part of the pituitary gland to avoid disrupting hormonal functions. The pituitary gland regulates numerous essential hormonal processes within the body, and its damage could lead to endocrine disorders, such as hypopituitarism, which may necessitate hormonal replacement therapy. Another limitation is related to the study’s small sample size (60 patients) from a single center, which limits its statistical power and generalizability. Taking also into account the contradictory findings of our study and those of Zheng et al. [14] concerning the presence of hrHPV in PITNETs, as well as the prognostic significance of this infection, further multi-center studies with larger sample sizes are necessary to validate and reinforce the study’s conclusions.

## 5. Conclusions

In the group of pituitary adenomas studied for the first time in Poland, the presence of transcriptionally active HPV infections from the high-risk subgroup (especially HPV16) was detected in a significant proportion of tumors. Our results suggest that this type of infection may play a role in the invasiveness of pituitary adenomas. Non-invasive pituitary adenomas have a higher prevalence of transcriptionally active high-risk HPV infections (including HPV16 in particular) as compared to invasive tumors. Positive infection was associated with a significantly lower probability of tumor invasiveness, measured on both the Knosp and Hardy scales. Further studies are needed to confirm the prevalence of transcriptionally active HPV infections in pituitary adenomas and to elucidate the role of these infections in pituitary tumorigenesis.

## Figures and Tables

**Table 1:** Patients’ clinical characteristics.

Characteristic	No.	Percent	Missing
**All patients**	60	100.0	
**Age [years]**			
≥61.0 *	30	50.0	
<61.0	30	50.0	
**Gender**			
Male	35	58.3	
Female	25	41.7	
**Histopathology**			
Gonadotroph	37	61.7	
Corticotroph	8	13.3	
Null-cell adenoma	5	8.3	
Multiple synchronous PITNETs	2	3.3	
Lactotroph	2	3.3	
Immature PIT-1-lineage PITNETs	3	5.0	
Thyrotroph	1	1.7	
Plurihormonal PITNETs	2	3.3	
**Knosp scale**			
1 + 2	25	43.1	
3 + 4	33	55.9	2
**Hardy scale**			
1 + 2	10	17.2	
3 + 4	48	87.5	2
**Hormonal activity**			
Yes	10	16.7	
No	50	83.3	
**PIT-1 overexpression**			
Yes	11	18.3	
No	49	81.7	
**TPIT overexpression**			
Yes	9	15.5	
No	49	84.5	2
**SFF-1 overexpression**			
Yes	43	71.4	
No	15	25.9	2
**AP [mm**] **			
≥20.0 *	25	42.4	
<20.0	34	57.6	1
**ML [mm**] **			
≥25.0 *	26	44.1	
<25.0	33	55.9	1
**CC [mm]** **			
≥23.0 *	28	47.5	
<23.0	31	52.5	1
**Max. tumor diameter [mm]** **			
≥26.0 *	28	47.5	
<26.0	31	52.5	1
**Tumor volume [cm^3^]**			
≥5.2 *	26	45.6	
<5.2	31	54.4	3

* Median level; ** one patient had only CT scan of the head before surgery; AP: maximum diameter in the anterior–posterior direction, ML: maximum diameter in the perpendicular direction, that is, the medio-lateral direction, CC: maximum diameter in the cranio-caudal direction.

**Table 2:** Demographic, clinical, and histopathological participant characteristics across the Knosp and Hardy scales.

Characteristics	Knosp Scale				*p*-Value	Hardy Scale				*p*-Value
	Non-Invasive		Invasive			Non-Invasive		Invasive		
	No.	Percent	No.	Percent		No.	Percent	No.	Percent	
**Age [years]**										
≥61	12	41.4	17	58.6	0.791	5	17.2	24	82.8	1.000
<61	13	44.8	16	55.2		5	17.2	24	82.8	
**Gender**										
Male	16	48.5	17	51.5	0.342	6	18.2	27	81.8	0.827
Female	9	36.0	16	64.0		4	16.0	21	84.0	
**Histopathology**										
Gonadotroph	16	43.2	21	56.8	0.642	5	13.5	32	86.5	0.072
Corticotroph	3	42.9	4	57.1		2	28.6	5	71.4	
Null-cell adenoma	2	50.0	2	50.0		0	0.0	4	100.0	
Multiple synchronous PITNETs	1	50.0	1	50.0		0	0.0	2	100.0	
Lactotroph	2	100.0	0	0.0		2	100.0	0	0.0	
Immature PIT 1-lineage PITNETs	1	33.3	2	66.7		1	33.3	2	66.7	
Thyrotroph	0	0.0	1	100.0		0	0.0	1	100.0	
Plurihormonal PITNETs	0	0.0	2	100.0		0	0.0	2	100.0	
**Hormonal activity**										
Yes	4	40.0	6	60.0	0.827	3	30.0	7	70.0	0.475
No	21	43.7	27	56.3		7	14.6	41	85.4	
**PIT-1 overexpression**										
Yes	3	30.0	7	70.0	0.358	3	30.0	7	70.0	0.475
No	22	45.8	26	54.2		7	14.6	41	85.4	
**TPIT overexpression**										
Yes	3	42.9	4	57.1	1.000	2	28.6	5	71.4	0.336
No	21	42.9	28	57.1		7	14.3	42	85.7	
**SFF1 overexpression**										
Yes	17	40.5	25	59.5	0.875	5	11.9	37	88.1	0.378
No	6	42.9	8	57.1		3	21.4	11	78.6	
**AP [mm]**										
≥20 *	6	25.0	18	75.0	0.019	0	0.0	24	100.0	0.003
<20	19	55.9	15	44.1		10	29.4	24	70.6	
**ML [mm]**										
≥25.0 *	7	28.0	18	72.0	0.043	0	0.0	25	100.0	0.002
<25.0	18	54.6	15	45.4		10	30.3	23	69.7	
**CC [mm]**										
≥23.0 *	8	29.6	19	70.4	0.053	1	3.7	26	96.3	0.011
<23.0	17	54.8	14	54.2		9	29.0	22	71.0	
**Max tumor diameter [mm]**										
≥26.0 *	9	32.1	19	67.9	0.103	1	3.6	27	96.4	0.008
<26.0	16	53.3	14	46.7		9	30.0	21	70.0	
**Tumor volume [cm^3^]**										
≥5.2 *	8	28.6	20	71.4	0.022	1	3.6	27	96.4	0.006
<5.2	17	58.6	12	41.4		9	31.0	20	69.0	

* Median level; AP: maximum diameter in the anterior–posterior direction, ML: maximum diameter in the perpendicular direction, that is, the medio-lateral direction, CC: maximum diameter in the cranio-caudal direction.

**Table 3:** The occurrence of specific HPV genotypes in the group of 60 PITNETs.

HPV Genotype	No.	Percent
**DNA of high-risk HPV**		
All hrHPV	13	21.7
Single infection of HPV16	7	53.8
Infection of HPV16 + one or more of the following genotypes: HPV31, 33, 35, 39, 45, 51, 52, 56, 58, 59, 66, 68	1	7.7
Infection of HPV18 + one or more of the following genotypes: HPV31, 33, 35, 39, 45, 51, 52, 56, 58, 59, 66, 68	1	7.7
Infection only with one or more of the following genotypes: HPV31, 33, 35, 39, 45, 51, 52, 56, 58, 59, 66, 68	4	30.8
**DNA of low-risk HPV**		
All lrHPV	26	43.3
Infection with HPV6 and/or HPV11 only	2	7.7
Infection with one or more of the following genotypes: HPV40, 42, 43, 44, 55, 83 only	4	15.4
Infection with one or more of the following genotypes: HPV26, 53, 67, 70, 73, 82 only	14	53.9
Infection with HPV6 and/or HPV11 and infection with one or more of the following genotypes: HPV40, 42, 43, 44, 55, 83	3	11.5
Infection with HPV6 and/or HPV11 and infection with one or more of the following genotypes: HPV26, 53, 67, 70, 73, 82	3	11.5

**Table 4:** Correlations between the presence of high-risk HPV DNA, P16 immunoexpression, and transcriptionally active viral infection, and demographic, clinical, and histopathological features of 60 patients with PITNETs.

Characteristic	Hr HPV DNA Positivity					P16 Overexpression					Transcriptionally Active hrHPV Infection				
	Yes		No		*p*-Value	Yes		No		*p*-Value	Yes		No		*p*-Value
	No.	Percent	No.	Percent		No.	Percent	No.	Percent		No.	Percent	No.	Percent	
**All patients**	13	21.7	47	78.3		39	65.0	21	35.0		11	18.3	49	81.7	
**Age [years]**															
≥61 *	6	20.0	24	80.0	0.754	19	63.3	11	36.7	0.787	4	13.3	26	86.7	0.317
<61	7	23.3	23	76.7		20	66.7	10	33.3		7	23.3	23	76.7	
**Gender**															
Male	6	17.1	29	82.9	0.314	22	62.9	13	37.1	0.681	5	14.3	30	85.7	0.338
Female	7	28.0	18	72.0		17	68.0	8	32.0		6	24.0	19	76.0	
**Histopathology**															
Gonadotroph	6	16.2	31	83.8	0.772	23	62.2	14	37.8	0.743	5	13.5	32	86.5	0.604
Corticotroph	2	25.0	6	75.0		6	75.0	2	25.0		1	12.5	7	87.5	
Null-cell adenoma	1	20.0	4	80.0		4	80.0	1	20.0		1	20.0	4	80.0	
Multiple synchronous PITNETs	1	50.0	1	50.0		1	50.0	1	50.0		1	50.0	1	50.0	
Lactotroph	1	50.0	1	50.0		1	50.0	1	50.0		1	50.0	1	50.0	
Immature PITT-lineage PITNETs	1	33.3	2	66.7		1	33.3	2	66.7		1	33.3	2	66.7	
Thyrotroph	0	0.0	1	100.0		1	100.0	0	0.0		0	0.0	1	100.0	
Plurihormonal PITNETs	1	50.0	1	50.0		2	100.0	0	0.0		1	50.0	1	50.0	
**Hormonal activity**															
Yes	4	40.0	6	60.0	0.123	8	80.0	2	20.0	0.276	4	40.0	6	60.0	0.052
No	9	18.0	41	82.0		31	62.0	19	38.0		7	14.0	43	86.0	
**Knosp scale**															
1 + 2	10	40.0	15	60.0	0.005	16	64.0	9	36.0	0.977	9	36.0	16	64.0	0.004
3 + 4	3	9.1	20	90.0		21	63.6	12	36.4		2	6.1	31	93.4	
**Hardy scale**															
1 + 2	5	50.0	5	50.0	0.021	8	80.0	2	20.0	0.241	5	50.0	5	50.0	0.006
3 + 4	8	16.7	40	83.3		29	60.4	19	39.6		6	12.5	42	87.5	
**PIT-1 overexpression**															
Yes	3	27.3	8	72.7	0.617	7	63.6	4	36.4	0.916	3	27.3	8	72.7	0.397
No	10	20.4	39	79.6		32	65.3	17	34.7		8	16.3	41	83.7	
**TPIT overexpression**															
Yes	2	22.2	7	77.8	0.988	7	77.8	2	22.2	0.400	1	11.1	8	88.9	0.513
No	11	22.5	38	77.5		31	63.3	18	36.7		10	20.4	39	79.6	
**SFF-1 overexpression**															
Yes	8	18.6	35	81.4	0.507	27	62.8	16	37.2	0.460	7	16.3	36	83.7	0.743
No	4	26.7	11	73.3		11	73.3	4	26.7		3	20.0	12	80.0	
**AP {mm]**															
≥20 *	3	12.0	22	88.0	0.111	15	60.0	10	40.0	0.544	1	4.0	24	96.0	0.013
<20	10	29.4	24	70.6		23	67.6	11	32.4		10	29.4	24	70.6	
**ML [mL]**															
≥25.0 *	3	11.5	23	88.5	0.084	16	61.5	10	38.5	0.683	2	7.7	24	92.3	0.055
<25.0	10	30.3	23	69.7		22	66.7	11	33.3		9	27.3	24	72.7	
**CC [mm]**															
≥23.0 *	3	10.7	25	89.3	0.046	16	57.1	12	42.9	0.269	2	7.1	26	92.9	0.031
<23.0 *	10	32.3	21	67.4		22	71.0	9	29.0		9	29.0	22	71.0	
**Max tumor diameter [mm]**															
≥26.0 *	3	10.3	26	89.7	0.033	17	58.6	12	41.4	0.361	2	6.9	27	93.1	0.023
<26.0	10	33.3	20	66.7		21	70.0	9	30.0		9	30.0	21	70.0	
**Tumor volume [cm^3^]**															
≥5.2 *	3	10.7	25	89.3	0.032	17	60.7	11	39.3	0.514	2	7.1	26	92.9	0.022
<5.2	10	34.5	19	65.5		20	69.0	9	31.0		9	31.0	20	69.0	

* Median level; AP: maximum diameter in the anterior–posterior direction, ML: maximum diameter in the perpendicular direction, that is, the medio-lateral direction, CC: maximum diameter in the cranio-caudal direction.

**Table 5:** Radiological and immunohistochemical characteristics of pituitary tumors positively expressing transcriptionally active hrHPV infection.

Age [Years]	Gender	Type of PITNET	Knosp Scale	Hardy Scale	Hormonal Activity	PIT-1 Overexpression	TPIT Overexpression	SFF-1 Overexpression	AP [mm]	ML [mm]	CC [mm]	Volume [cm^3^]
64	M	Multiple synchronous	4	4E	Yes	No	No	Yes	40.0	37.0	45.0	33.0
43	F	Lactotroph	1	2A	Yes	No	Yes	No	12.0	23.0	10.0	1.4
62	F	Immature PIT-1	1	2A	Yes	No	No	Yes	12.5	20.0	14.0	1.6
51	M	Gonadotroph	2	3D	No	No	Yes	No	19.5	27.0	28.0	6.5
57	F	Null-cell adenoma	2	2B	No	No	No	No	14.0	14.0	17.5	2.1
53	F	Gonadotroph	1	2B	No	No	No	Yes	18.0	20.0	21.5	5.0
74	M	Gonadotroph	2	2B	No	No	No	Yes	18.5	19.0	22.5	3.2
73	M	Gonadotroph	1	2B	No	No	No	Yes	14.5	19.0	16.0	2.1
38	F	Corticotroph	1	1A	No	Yes	No	No data	4.5	5.5	4.5	0.8
58	M	Gonadotroph	1	2A	No	No	No	Yes	13.0	21.0	13.0	1.2
52	F	Mature PIT1	3B	4D	No	No	Yes	Yes	17.0	18.0	23.0	4.0

M: male, F: female, AP: maximum diameter in the anterior–posterior direction, ML: maximum diameter in the perpendicular direction, that is, the medio-lateral direction, CC: maximum diameter in the cranio-caudal direction.

**Table 6:** Crude and adjusted models of multivariate logistic regression analyses. Modeled probability: tumor invasiveness on the Knosp and Hardy scales. Probability: tumor invasiveness on the Knosp and Hardy scales.

Invasiveness	Characteristics	Crude OR		Adjusted OR (Full Model) *		Adjusted OR (Final Model) **	
		OR (95% CI)	*p*-Value	OR (95% CI)	*p*-Value	OR (95% CI)	*p*-Value
Knosp scale	Transcriptionally active hrHPV infection	0.11 (0.02; 0.60)	0.01	0.03 (0.00; 0.48)	0.01	0.11 (0.02; 0.58)	0.01
				Wald test, *p*-value = 0.32		Wald test, *p*-value = 0.01	
				AIC = 75.7		AIC = 71.7	
				Cox–Snell R^2^ = 0.23		Cox–Snell R^2^ = 0.14	
				Nagelkerke R = 0.31		Nagelkerke R = 0.19	
Hardy scale	Transcriptionally active hrHPV infection	0.14 (0.03; 0.64)	0.01	0.06 (0.01; 0.44)	0.01	0.12 (0.02; 0.56)	0.01
				Wald test, *p*-value = 0.20		Wald test, *p*-value = 0.01	
				AIC = 52.9		AIC = 46.2	
				Cox–Snell R^2^ = 0.17		Cox–Snell R^2^ = 0.12	
				Nagelkerke R = 0.29		Nagelkerke R = 0.21	

* Adjusted by age, sex, hormonal activity, PIT-1, and TPIT expression; ** the results of the stepwise backward multivariate logistic regression analysis.

## Data Availability

Reports concerning qPCR reaction and P16 immunostaining are available on reasonable request from the corresponding author.
